# Laminar functional magnetic resonance imaging in vision research

**DOI:** 10.3389/fnins.2022.910443

**Published:** 2022-10-04

**Authors:** Pinar Demirayak, Gopikrishna Deshpande, Kristina Visscher

**Affiliations:** ^1^Civitan International Research Center, University of Alabama at Birmingham, Birmingham, AL, United States; ^2^Department of Neurobiology, University of Alabama at Birmingham, Birmingham, AL, United States; ^3^Department of Electrical and Computer Engineering, AU MRI Research Center, Auburn University, Auburn, AL, United States; ^4^Department of Psychological Sciences, Auburn University, Auburn, AL, United States; ^5^Alabama Advanced Imaging Consortium, Birmingham, AL, United States; ^6^Center for Neuroscience, Auburn University, Auburn, AL, United States; ^7^School of Psychology, Capital Normal University, Beijing, China; ^8^Key Laboratory of Learning and Cognition, Capital Normal University, Beijing, China; ^9^Department of Psychiatry, National Institute of Mental Health and Neurosciences, Bangalore, India; ^10^Centre for Brain Research, Indian Institute of Science, Bangalore, India

**Keywords:** laminar fMRI, vision research, cortical layers, attention, perception

## Abstract

Magnetic resonance imaging (MRI) scanners at ultra-high magnetic fields have become available to use in humans, thus enabling researchers to investigate the human brain in detail. By increasing the spatial resolution, ultra-high field MR allows both structural and functional characterization of cortical layers. Techniques that can differentiate cortical layers, such as histological studies and electrode-based measurements have made critical contributions to the understanding of brain function, but these techniques are invasive and thus mainly available in animal models. There are likely to be differences in the organization of circuits between humans and even our closest evolutionary neighbors. Thus research on the human brain is essential. Ultra-high field MRI can observe differences between cortical layers, but is non-invasive and can be used in humans. Extensive previous literature has shown that neuronal connections between brain areas that transmit feedback and feedforward information terminate in different layers of the cortex. Layer-specific functional MRI (fMRI) allows the identification of layer-specific hemodynamic responses, distinguishing feedback and feedforward pathways. This capability has been particularly important for understanding visual processing, as it has allowed researchers to test hypotheses concerning feedback and feedforward information in visual cortical areas. In this review, we provide a general overview of successful ultra-high field MRI applications in vision research as examples of future research.

## Introduction

The human cortex is approximately 1–4.5 mm thick, with an overall average of approximately 2.5 mm (Fischl and Dale, 2000). The first cytoarchitectonic work by Brodmann (1909) showed that the human cortex is subdivided into six layers based on neuron morphology and differences in the spatial distribution of cells. Ultra-high field magnetic resonance imaging (MRI), unlike more typical MRI methods, can measure neural activity at the level of neocortical layers. Ultra-high field MRI provides direct interpretability of physiological changes and promises precise localization in applications of layer-specific functional MRI (fMRI). Advantages of layer-specific MRI include an increase in signal-to-noise ratio (SNR) and blood-oxygenation level dependent (BOLD) contrast that allows examination of layer-specific neuronal processing in scientific research.

**FIGURE 1:**
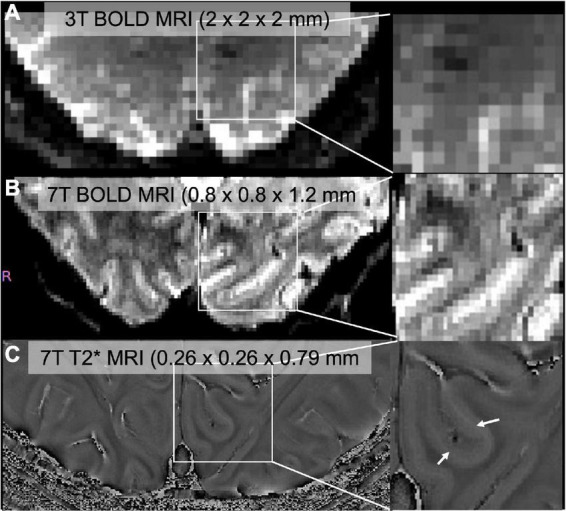
**(A)** Standard resolution BOLD image through V1 collected at 3T (Siemens Prisma), **(B)** high-resolution BOLD image collected at 7T (Siemens Magnetom), **(C)** high-resolution T2* image. Zoomed images were shown in the right panel. We can identify a middle layer that corresponds to the stria of gennari (white arrows) in 7T but not in 3T.

Lower magnetic field intensities (1.5T or 3T) for human brain imaging are limited in spatial resolution (Figure 1). Higher magnetic field strengths in human brain imaging have made examinations of the structural and functional organization of layers of the neocortex feasible. Human neocortical layers have distinct cytoarchitecture (Hevner, 2007), connectivity, and function (Finn et al., 2021). There are characteristic differences in axonal connections in ascending and descending pathways in the visual hierarchy: thalamo-cortical projections terminate in layer 4 (feedforward pathway) while descending pathway terminates mainly in superficial and deep layers (feedback pathway) (Rockland and Pandya, 1979; Maunsell and Van Essen, 1983; Felleman and Van Essen, 1991). Many layer-specific studies investigate the modulation of balance of feedback or feedforward influences on the primary visual cortex. Applications of ultra-high field MRI in vision studies include object perception, attention, and multisensory integration. Here we will review successful applications of ultra-high field MRI in vision research.

## Vascular physiology and neural activity

Magnetic resonance imaging at ultra-high field benefits from a high SNR that leads to high spatial resolution and high contrast-to-noise ratio (CNR); these advantages enable examination of the effect of vascular architecture on neuronal activity (Logothetis et al., 2001). BOLD contrast is generated by changes in deoxyhemoglobin (Drew, 2019). Deoxygenated hemoglobin concentration is a result of cerebral blood volume (CBV), cerebral blood flow (CBF), and oxygen metabolism following neuronal excitation (van Zijl et al., 1998; Uludağ et al., 2009). The BOLD signal is sensitive to these changes. Differences in laminar distribution of the BOLD signal among cortical layers was investigated in cats and primates in 4.7T, 7T, and 9.4T magnetic fields (Zhao et al., 2004; Goense and Logothetis, 2006; Goense et al., 2007). A common finding from these studies showed that Gradient Echo (GE) fMRI signals peak at surface vessels, near layer 1, whereas Spin Echo (SE) fMRI signals peak at layer 4. Vascular contributions to the BOLD signal from these layers should be considered in any layer-specific fMRI research, depending on the scanning parameters.

Recent papers have argued about the degree to which high field MRI techniques can be biased toward an increase in BOLD signal in the superior layers of the cortex due to patterns of the vasculature (Huber et al., 2018). Thus, the possibility of vasculature architecture-based artifact involvement should be considered and any vasculature-based artifact should be omitted from the data for layer-specific vision experiments. One recent work reported a new cortical depth-dependent model of the BOLD response based on mass-balance principles, which takes the effect of ascending (and pial) veins on the cortical BOLD responses explicitly into account (Havlicek and Uludağ, 2020). Such extensions to the original balloon model of the hemodynamic response function are capable of modeling signal generation at the laminar level by taking local vascular physiology into account. Such a model could potentially be used to deconvolve the contributions of laminar specific vasculature to the BOLD signal so that the deconvolved data can be treated as free of vasculature-based artifacts.

VAscular-Space Occupancy (VASO) fMRI is a technique to detect brain activation based on the changes in CBV as opposed to BOLD fMRI which is based on blood-oxygenation (Lu and van Zijl, 2012). VASO fMRI takes advantage of the T1 difference between the blood and surrounding tissue to purify the neuronal activation signal. Both BOLD imaging and VASO fMRI techniques have advantages and disadvantages. Comparison of VASO using gradient echo 3D-EPI and BOLD using 3D-gradient and spin echo (GRASE) show similar specificity and sensitivity during a motor task but the combination of these techniques did not necessarily demonstrate better sensitivity and specificity (Beckett et al., 2020). The main benefit of the VASO fMRI compared to BOLD fMRI is providing excellent localization, unlike the BOLD, signals did not extend to distant regions when oxygenation of the blood changes in cat brain (Jin and Kim, 2008). Another advantage of using VASO is improving quantification of extravascular BOLD signal (Lu and van Zijl, 2012). Thus, combining BOLD and VASO allows a better understanding of metabolic and hemodynamic changes during neural activity. Although there are advantages of the VASO technique there are also disadvantages of VASO. One of the disadvantages of VASO compared to GE-BOLD is reduced sensitivity. CNR of the VASO is lower than BOLD, between 20–33% (Lu et al., 2003) and 60% of the BOLD (Huber et al., 2014). Also, VASO has reduced imaging efficacy due to the acquisition delay that is required for T1 contrast (Huber et al., 2018). Hence, VASO sequences have longer TR when compared to BOLD images. The main disadvantage of VASO is having lower temporal resolution. Quantitative CBV-based fMRI based on 3D Echo Planar Imaging (EPI) could overcome these limitations of BOLD and outperforms GE BOLD and 2D-EPI (Huber et al., 2018).

The vast majority of fMRI studies use T2*-weighted gradient echo planar imaging (GE-EPI) since it has high SNR, however ultra-high magnetic field MRI makes usage of spin-echo echo planar imaging (SE-EPI) possible by minimizing veinous artifacts (Park et al., 2021). However, the application of inner-volume-based SE-EPI is limited to a flat piece of the cortex (Duong et al., 2003). Thus, it is challenging to apply beyond primary visual areas (Park et al., 2021). 3D-GRASE imaging with inner volume selection helps to overcome this problem. De Martino et al. (2011)colleagues showed that 3D-GRASE improves specificity in human laminar fMRI data and the sequence is less sensitive to contributions from the large veins in superficial layers due to high specificity. The GRASE technique helps to reduce venous contributions to neural activity in the early visual cortex in vision studies.

Another technique to identify CBF is arterial spin labeling (ASL) which allows quantification of the CBF by using magnetically labeled arterial blood water as an endogenous tracer (Williams et al., 1992). It is helpful to provide information related to blood flow to reduce artifacts in neuronal activation signals. However, ASL is also limited with a low SNR, longer scanning times, and limited spatial coverage. A pulsed ASL (PASL) technique utilizes a simultaneous multi-slice approach and allows for better temporal and spatial resolution and better SNR (Ivanov et al., 2017). VASO, GRASE, and ASL techniques help to reduce venous contributions to neural activity in the cortex by providing better specificity.

Ultra-high field functional MRI has advantages compared to lower magnetic field strengths. The amplitude of BOLD response increases linearly with field strength (van der Zwaag et al., 2009), and it has been shown that microvascular contributions were relatively large due to diminished intravascular signals from large venous vessels (Yacoub et al., 2001; Uludağ et al., 2009; Kim and Ogawa, 2012). However, high magnetic field strength enables better identification of the venous contribution by implementing susceptibility-weighted MR venography (Koopmans et al., 2008) or physiological noise (van der Zwaag et al., 2015) compared to lower magnetic strengths. It allows obtaining artifact-free neural activity and leads to larger more tissue-specific functional responses.

Another advantage of using ultra-high field MRI is providing a higher SNR (Moeller et al., 2010). The relative benefits of GE and SE are open to debate in the high magnetic field imaging area. While GE provides high temporal SNR that is sensitive to both macro and microvascular signals, SE is more specific to microvasculature but suffers from lower temporal SNR (Stanley et al., 2021). Applications of higher magnetic field imaging techniques in scientific research including vision research are very sensitive to parameter choice and optimization of the parameters to be able to accurately interpret the results.

## Data processing in laminar functional magnetic resonance imaging studies

While T1-weighted images tend to keep veridical representations of the relative sizes and shapes of anatomical structures, EPI images are more vulnerable to distortions because of the relatively low bandwidth along the specific phase-encoding direction (Polimeni et al., 2018). For this reason, incorporating high resolution T1 weighted data to high resolution EPI data requires geometric correspondence between anatomical and functional data. In high resolution data, usually EPI images are used as reference images for anatomical-functional data registration. Although image reconstruction for high-resolution submillimeter T1 weighted images recently became available (Zaretskaya et al., 2018), EPI-registered T1-weighted image-based measurements such as morphometric analyses are not suitable. Moreover, improvements in EPI acquisition techniques allow for the removal of distortions in T2* weighted before T1 weighted data registration to some degree. Distortion correction including magnetic field inhomogeneities is critical to achieve accurate registration of anatomical and functional data. Distortions are worse in ultra-high magnetic fields compared to lower magnetic fields. Thus, fMRI preprocessing steps should be adapted to high-resolution data.

Usually, preprocessing steps include magnetic field inhomogeneity corrections and slice-timing corrections. Slice-timing correction is often critical for multi-band acquisitions that are used to increase temporal resolution or spatial coverage (Feinberg et al., 2010; Feinberg and Setsompop, 2013; Barth et al., 2016). In addition, the motion correction step includes the estimation of rigid-body head motion and correction for that motion. To retain data quality, usually a reference image is chosen and each image is rotated and translated to fit the reference image. Since some blurring and resolution loss is inevitable during motion correction, it can be accepted if motion occurs occasionally and within certain limits (Polimeni et al., 2018). Registrations between anatomical and functional data can be performed by using non-linear transformations which are preferable for ultra-high field fMRI data.

**FIGURE 2:**
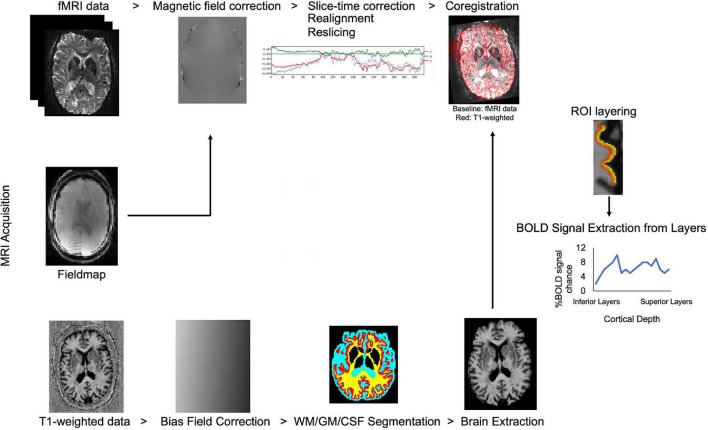
Flow chart of laminar fMRI processing steps. Potential MRI sequences used in laminar fMRI studies are (from top to bottom of image) fMRI, field map, and T1 weighted sequences. Standard preprocessing steps for the fMRI data are listed left to right. T1 weighted data is corrected for bias field, segmented for white matter, gray matter, and cerebrospinal fluid and brain is extracted for better results in coregistration to the BOLD fMRI data. Regions-of-interest are created in the desired area and layered. The BOLD signal can be extracted from laminar layers.

Due to technological advancements in ultra-high field MRI, allowing high resolution data collection, identification of cortical layers has become feasible in human studies. Processing steps in laminar fMRI studies were summarized in Figure 2. Advanced data processing approaches enable cortical layering from the lower magnetic fields (3T) as well. These approaches overcome some of the limitations of ultra-high field MRI such as limited field-of-view (FOV) and allow a bigger head coil usage. Larger head coils can be beneficial for participant comfort and to fit ancillary equipment, for example, to measure simultaneous EEG activity. Scheeringa et al. (2016) showed layer-specific EEG powers during feedback and feedforward information processing in early visual cortex by using high resolution (0.75 mm isotropic) 3T MR images. They collected both lower resolution (3 mm isotropic, functional; 1 mm isotropic anatomical) and higher resolution (0.75 mm isotropic) for both functional and anatomical) data. Data processing was benefited from advantages of using both lower and higher resolution images. Moreover, Shamir et al. (2019) proposed a different data processing method, they showed that by using a low-resolution 3T MRI echoplanar imaging inversion recovery protocol multiple T1 relaxation time components per voxel can be extracted and assigned to different types of brain tissue. Their approach to layer their 3T EPI data, uses volumetric sampling of virtual spheres dispersed throughout the entire cortical space. Laminar fMRI is also feasible in lower magnetic strengths by using appropriate data processing approaches.

Another important vulnerability of high-resolution fMRI data is partial voluming. Partial voluming is a phenomenon where a voxel’s volume is partially filled with tissue of one type, and partially filled with another type of tissue, causing a signal that may be reflective of multiple effects. When attempting to dissociate signals from different layers of cortex, partial voluming is inevitable unless the size of the voxel is less than or equal to the width of the layer. Such small voxels are largely not achievable in humans, except in certain special circumstances such as line scanning, which would sacrifice other important requirements such as coverage (Raimondo et al., 2021). Therefore, researchers must currently accept some partial voluming and loss of layer specificity in exchange for better coverage and temporal resolution. Previous data has demonstrated that even with this partial voluming, we have sensitivity to layer specific neural processes. De Martino et al. (2011) investigated the partial volume effect in higher resolutions for resting-state network analysis with whole brain coverage. They adapted conventional fMRI analysis methods for high-resolution fMRI data including resampling at 1 mm. Using independent component analysis, smaller voxel volumes resulted in reduced partial volume effects. These allow better localization and identification of the resting state networks.

In the future, we believe that improvements in technology will make the terms of this tradeoff between loss of a layer specificity and better coverage and temporal resolution favorable. However, for now, one has to pick parameters important for the given application and proceed to optimize that. If lack of partial voluming is the most important criteria for a given application (such as investigating spatial and orientation tuning), then the option of line scanning exists if restricted coverage is acceptable.

Overall, despite great advancements of ultra-high resolution fMRI data processing phase is critical to overcome some of the limitations, such as higher magnetic field inhomogeneity, partial voluming or limited FOV.

## Layer-selective functional magnetic resonance imaging responses in lateral geniculate nucleus and superior colliculus

Lateral geniculate nucleus (LGN) and superior colliculus (SC) have multiple ocular dominance layers relaying visual information from the retina to V1. LGN and SC receive direct input from the retina making them part of the earliest stages in the visual hierarchy. They play an important role in visual information processing, binocular rivalry and visual attention studies (Chen et al., 1998; Kastner et al., 2004; Schneider et al., 2004). Also, laminar studies in LGN and SC allow for differentiating laminar activity in early-stage glaucoma and normal controls (Zhang et al., 2016). Zhang et al. (2016) showed a reduction of BOLD response to transient achromatic stimuli relative to sustained chromatic stimuli in the magnocellular layers of LGN and superficial layers of SC in early-stage glaucoma patients. Similarly, BOLD responses in LGN were reduced in the amblyopic eye compared to the normal eye (Hess et al., 2009). These applications of layer-specific FMRI in subcortical structures in patients with eye diseases provide a great starting point for cortical laminar fMRI vision studies since they represent altered retinal inputs to the primary visual cortex.

## Forward, backward, and lateral connections within the cortex

**FIGURE 3:**
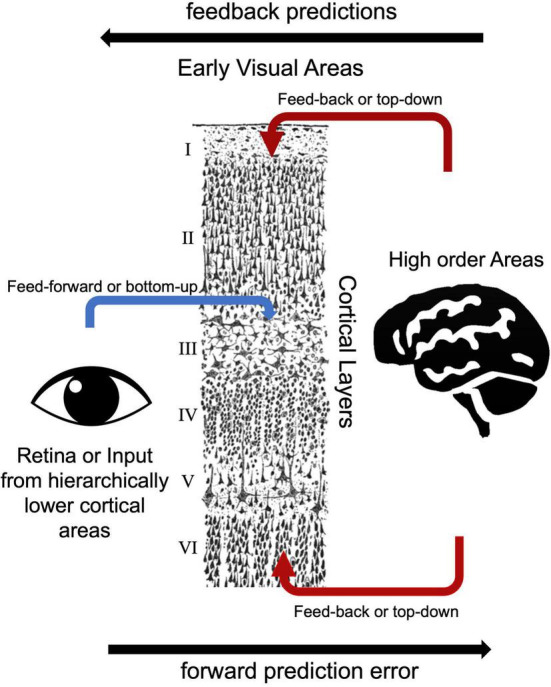
Bottom-up and top-down visual processing pathways to the early visual areas. The drawing of cortical layers is adapted from y Cajal (1899).

Based on the laminar origin and destination of connections within the visual processing pathway, researchers can distinguish feedback and feed-forward connections (Felleman and Van Essen, 1991). V1 area receives main feed forward inputs from the LGN of the thalamus (Angelucci and Bressloff, 2006). Pioneering electrode-based invasive research showed feedforward axonal connections from the LGN to the primary visual cortex (V1) terminate in layer 4 in the macaque model (Hubel and Wiesel, 1972). Similarly, human studies showed that feedforward projections target layer 4 (Hubel and Wiesel, 1972; Blasdel and Fitzpatrick, 1984; Michalareas et al., 2016; Figure 3). Lateral connections among V1 layers were identified in layers 2/3, 4B/upper 4Cα, and 5/6 predominantly terminate in upper layer 4 and superficial layers (Rockland and Pandya, 1979; Rockland and Lund, 1983). Feedback connections arise from superficial and/or deep layers and terminate in outside of layer 4 (Felleman and Van Essen, 1991; Angelucci and Bressloff, 2006). Self et al. (2013) examined cortical layer-specific activity during visual perception, and these layer-specific effects correspond well to the patterns of axonal connections among feedforward, lateral, and feedback observed using multicontact depth electrodes (Self et al., 2013). Consistent with these invasive studies, ultra-high field MRI studies showed feedback responses in deep and superficial but not middle cortical layers in the visual cortex (Muckli et al., 2015; Kok et al., 2016; Scheeringa et al., 2016). In addition to electrophysiology and task-based fMRI studies, resting state fMRI studies also investigate functional connections among early visual areas. Raemaekers et al. (2014) showed high functional connectivity between the same topographic locations in the field maps of V1, V2, and V3 and high functional connectivity between iso-eccentric locations in the same visual area by using ultra-high field MRI. The distinct nature of feedback, lateral and feedforward connections are thought to serve distinct functional roles (Self et al., 2013). Components of vision, including boundary detection, visual predictions, and illusory visual perception resulted in distinct laminar activity, consistent with their reliance on different axonal connections between visual cortex layers.

## Effect of attention on visual areas

Directing attention toward the most salient parts of a visual object or scene affects the processing of sensory stimuli. Visual perception does not solely depend on sensory stimuli, the brain also uses prior knowledge about the environment. Models of perception assume that the brain adapts predictions about sensory stimuli based on Bayesian inferential principles (Friston, 2005, 2009). Predictions influence perception *via* top-down signals by exploiting information about prior probabilities (Summerfield and de Lange, 2014; Schröger et al., 2015). Attention prioritizes the processing of specific sensory information that is suitable for current goals (Summerfield and Egner, 2009). Studies have shown that V1 activity is not limited to retinal sensory input processing, it can also be driven by attention-related activity (Kastner et al., 1999; Somers et al., 1999; Giesbrecht et al., 2006; Lawrence et al., 2018) and task-related information (Masuda et al., 2008, 2010, 2020). An fMRI study from Masuda et al. (2008) showed that patients who lost their central vision had greater stimulus-driven activity in the area of V1 corresponding to the scotoma than their matched healthy controls, but only when they attended to the stimulus, not when the stimulus was unattended. Also, experiments in participants with healthy vision have shown that attention to an area in the visual field involves top-down control from cortical “control” networks, preferentially affecting activity in the sections of the visual cortex that process attended parts of the visual field (Silver et al., 2007; Lauritzen et al., 2009). Attentional modulation involves networks of cortical areas including frontal eye fields, dorsomedial prefrontal, and posterior parietal cortices (Kastner and Ungerleider, 2000; Corbetta and Shulman, 2002). Effects of attentional modulation on the V1 were shown in deeper and superficial layers where the feedback projections terminate in both macaques (Anderson and Martin, 2009) and humans (Muckli et al., 2015; Kok et al., 2016). A study that combined ultra-high field MRI and EEG showed BOLD responses in superficial and deep layers of V1 that correlated with EEG oscillations as a response to an attention-requiring task (Scheeringa et al., 2016). Similarly, Lawrence et al. (2018) showed that item-specific visual memory signals evoke activity in both deep and superficial layers of V1 only but not layer-specific in V2 and V3. A more recent study from Scheeringa et al. (2022) found that alpha band oscillations relate to feedback-related laminar fMRI-based connectivity between deep-to-superficial layers of early visual areas. Lamina-specific attentional modulation of early visual areas is a growing area of vision research and successful applications of ultra-high field imaging to this area provide important details about the mechanisms of attentional modulation in visual areas.

## Luminance contrast processing in cortex layers

Cortical areas at different levels of the visual hierarchy show different contrast sensitivity in humans (Boynton et al., 1999; Marquardt et al., 2018). Due to the hierarchical nature of visual processing, neurons in V1 are involved in processing simple luminance contrasts whereas extrastriate cortical areas are more involved in processing complex visual features (Maunsell and Newsome, 1987; Hochstein and Ahissar, 2002). Marquardt et al. (2018) showed contrast sensitivity difference between V1 and V2 however they did not find evidence for variations in contrast sensitivity as a function of cortical layers. A more recent study identified a linear amplitude scaling across cortical depth in V1, higher luminance contrast elicit higher BOLD response amplitude in superficial layers in V1 (van Dijk et al., 2020). Another recent study by Lawrence et al. (2019) found that fMRI responses in early visual areas were modulated by changes in stimulus contrast and feature-based attention. Their results indicated that attentional processing is more involved in superficial layers whereas stimulus contrast change processing is more involved in middle layers (Lawrence et al., 2019). In addition, *in vivo* studies showed that sensitivity to visual stimulus features varies between layers of the visual cortex, indicating that while the layers are connected to each other, they are a part of separate information processing pathways within a visual hierarchy (Hubel and Wiesel, 1968; Alonso and Martinez, 1998; Martinez and Alonso, 2003). Also, a glucose-uptake study reported that stimuli with different contrast levels elicited activity in either magnocellular or parvocellular layers of LGN that have segregated projection to different layers of the striate cortex and still remain somewhat segregated in their projections to the extrastriate cortex in a monkey model (Tootell et al., 1988). Together, these examples show how laminar fMRI can be used to understand how contrast is processed throughout the ascending pathway from LGN to early visual areas.

## Object perception

In the classical perspective, visual processing from the retina follows two visual streams; ventral and dorsal pathways. Object perception involves cross-talk between these two streams (Peissig and Tarr, 2007; Konen and Kastner, 2008; Perry and Fallah, 2014). Since environmental stimuli are often not optimal, cross-talk between ventral and dorsal pathways in object perception is necessary to reconstruct the contours of an object from prior knowledge. Vision research experiments have shown that feedback from higher cortical levels, such as V4, IT, or MT can modify V1 responses (Hupé et al., 2001; Murray et al., 2004; Sillito et al., 2006; Sterzer et al., 2006; Huang et al., 2007). In studies of object perception, illusory contour stimuli have been used to investigate the filling-in of information *via* feedback from higher-order areas to lower-level visual information processing areas. Object perception studies showed illusory contour-related activity emerging first in the Lateral Occipital Cortex (LOC), then V2 and finally in V1, suggesting the BOLD response is driven by feedback inputs (Lee and Nguyen, 2001; Murray et al., 2002). A computational modeling paper indicated feedforward object recognition including cases of occluded and illusory images (Dura-Bernal et al., 2011). Layer-specificity in grouping features of an object was investigated; the study showed that circuits in V1 and V2 areas involving multiple layers are necessary for feedback, feedforward and horizontal interactions for perceptual grouping (Grossberg and Raizada, 2000). Investigations into the mechanisms of object perception benefit from the layer-specific information from ultra-high resolution fMRI because it can inform us about the role of feedback, feedforward, and horizontal connections in the visual processing of objects.

## Multisensory integration

In the human cortex, sensory areas interact with each other, for example, an auditory stimulus can elicit a hemodynamic response in the V1 (Campus et al., 2017; Petro et al., 2017), or circuits in V1 can control visuomotor behavior (Tang and Higley, 2020). Feedback projections between sensory information processing areas can modulate the activity in these areas (Felleman and Van Essen, 1991). These cortico-cortical feedback projections were traced in animal models indicating that there are direct projections from the primary auditory processing area (A1) to V1 but not vice versa and this projection is mainly from layer 5 of A1 to layer 1 of V1 (Ibrahim et al., 2016). Similarly, a recent paper from Gau et al. (2020) showed that multisensory interactions in auditory cortices were stronger in deeper cortical layers, while attentional influences were greatest at the surface layers in humans. These distinct depth-dependent profiles suggest that multisensory and attentional mechanisms regulate sensory processing *via* partly distinct circuitries (Gau et al., 2020). Similarly, Tang and Higley (2020) showed that layer 5 circuits of V1 plays a key role in visiomotor behavior control (Tang and Higley, 2020). Their 2-photon calcium imaging study on a mouse model indicated that layer 5 neurons in V1 strongly encode sensory and motor and sensory task information and this information is necessary for performance. Understanding the neural basis of multisensory integration is a potential application of ultra-high field MRI.

## Limitations of laminar functional magnetic resonance imaging

Although ultra-high field MRI has great advantages to image layer-specific cortical structure and activity, high magnetic field imaging also has several limitations. The biggest challenge is to increase the spatial resolution enough to image, interpret and measure artifact-free layer-specific BOLD response. BOLD response measures vascular signal changes, and thus it is restricted by the vascular architecture. GE BOLD is susceptible to venous-based artifacts on cortical layers (Uðurbil et al., 2003; Uludağ et al., 2009). These venous-based artifacts lead to increased signal strength toward the cortical surface (Koopmans et al., 2011; Muckli et al., 2015; Kok et al., 2016). Thus, BOLD activity on the superficial layers is more sensitive to vascular architecture-based artifacts. While interpreting data acquired using BOLD, the possible addition of this venous artifact should be considered.

Several different techniques have been used to overcome GE-BOLD related artifacts through the processing of data. Muckli et al. (2015) showed increased BOLD activity in superior layers of V1 as a result of top-down effects from high order cortical areas (Muckli et al., 2015). They excluded voxels with large receptive fields to omit the possibility of including venous-based artifacts. Kok et al. (2016) discussed the possible contribution of this vasculature-based artifact to increased BOLD activity in superficial layers in the previous literature. They assessed the interdependencies between layers by regressing out the signals from neighboring layers to show unique contributions from each layer (Kok et al., 2016). Also, as mentioned earlier, VASO fMRI is sensitive to activation-based changes in CBV and can be used to overcome vasculature-architecture based artifacts by differentiating blood and surrounding tissue (Lu and van Zijl, 2012). These studies indicate that ultra-high resolution fMRI can provide layer-specific cortical activity despite the possible inclusion of vasculature-based artifacts. A variety of different methods can be used in appropriate contexts to address these artifacts.

In vision research, functional connectivity measurement is sensitive to coupling dynamics of different brain regions that are involved in visual processing. Despite the significant gains in spatial resolution in BOLD and non-BOLD fMRI in the ultra-high fields, it is often difficult to maintain a larger field of view during the acquisitions. Increasing the imaging coverage with acceptable losses in temporal resolution is critical for functional connectivity measurements that are commonly used in vision research. One of the strategies that are used to extend the coverage area is using EPI with simultaneous multi-slice imaging by using multi-band excitations (Feinberg and Setsompop, 2013; Barth et al., 2016). Multi-band excitations in 2D were developed to cover larger brain areas with better SNR and temporal resolution, however spatial resolution of the acquisition is around 2.5 mm (Ivanov et al., 2017). The alternative to 2D multi-band imaging is 3D-EPI imaging as it allows whole brain coverage but spatial resolution is still around 1.5 mm isotropic voxels (Poser et al., 2010). Moving from conventional 2D slice-by-slice imaging to both multi-band imaging and highly accelerated 3D BOLD imaging allows submillimeter (0.75 mm) imaging with higher SNR and improved encoding efficiency (Setsompop et al., 2016). However, it is limited to use of partial-brain experiments due to the small field of view. It is easier to obtain a larger field of view with sub-mm voxels and acceptable temporal resolution for BOLD as compared to non-BOLD contrasts such as VASO. That said, ingenious placement of slices could still image all relevant areas without whole brain coverage, maintaining sub-mm voxels and a TR less than 2 s. For example, coronal, rather than the sagittal orientation of slices allows one to image most regions of the visual system including higher visual areas in a few slices without whole brain coverage, enabling investigation of layer-specific functional connectivity within the lower order and higher order visual networks. Similarly, appropriately tilted slices might enable simultaneous imaging of visual/parietal/temporal and frontal areas so as to enable imaging feedforward and feedback pathways within specific networks such as dorsal/ventral visual pathway or fronto-visual network. For now, such strategies seem to be the best bet for capturing a larger swathe of the cortex while still achieving spatial and temporal resolution goals. Thus, vision researchers should determine their scanning parameters by considering trade-offs of the sequences and their specific research questions.

## Conclusion

Ultra-high resolution MRI can identify laminar profiles of the human cortex, layer-specific hemodynamic responses, and functional communications between brain areas in more detail than previously available with MRI at lower magnetic field strengths. In this review, we described some recent successful applications of layer-specific fMRI techniques in vision research, including attention, luminance contrast processing, object perception, multisensory integration and summarized these applications with details of scanner parameters, their hypotheses and remaining questions in Table 1. Ultra-high field imaging is a precise method for elucidating hemodynamic response in cortical laminae and connection between brain areas to process visual information.

**TABLE 1:** Summary of recent laminar fMRI applications in vision research including parameters of the scan, hypothesis of the study, their findings, and remaining questions.

References	Sequence	Scanner	TR (seconds)	Resolution	Hypothesis	Findings	Remaining questions
Gau et al., 2020	2D GE single shot EPI	Magnetom 7T Siemens	3	0.75 mm^3^ iso	Modality-specific attention processing mostly happens in superficial cortical layers whereas visual influences on auditory cortices elicits activity in deep layers of the auditory cortex.	Multisensory interactions in auditory cortices are stringer in deep layers whereas attentional influences are stronger in superficial layers	Multisensory responses are mainly depend on stimulus salience and other input characteristics of the stimulus/how these factors influence laminar BOLD response profiles
Kok et al., 2016	3D GE-EPI	Magnetom 7T Siemens	3.408	0.8 mm^3^ iso	Feedback-mediated activity increase in V1 during the perception of illusory shapes should lead to a specific laminar activity profile that is distinct from the activity elicited by bottom-up stimulation	Top-down signals selectively activate deep layers of V1	More specificity on neural responses by considering not only amplitude but also information content using multivariate pattern analyses
Lawrence et al., 2018	T2*: 3D GE-EPI T2: HASTE	Magnetom 7T Siemens	3.408	0.8 mm^3^ iso	Feedback signals in early visual areas during visual working memory processed in deep and/or superficial layers but not in the middle layer	Item-specific visual working memory signals elicits activity in deep and superficial layers of V1 but all layers were activated in all layers in V2 and V3.	Designing a visual working memory task to target extrastriate cortex, involving the retrieval of more complex visual features such as angles, curves or whole objects.
Lawrence et al., 2019	3D GE-EPI	Magnetom 7T Siemens	3.408	0.8 mm^3^ iso	BOLD responses in early visual areas modulated by both bottom up and top-down regulations and these effects can be revealed by contrast and feature-based attention modulations, respectively.	Attentional processing is more involved in superficial layers whereas stimulus contrast change processing is more involved in middle layers of early visual areas.	Dynamic interactions between bottom-up and top-down processing in layers of the cortex can be investigated by simultaneous measurements over larger areas of cortex compared to EEG
Marquardt et al., 2018	3D GE-EPI	Siemens 7T	2.94	0.7 mm^3^ iso	Luminance contrast elicits activity in middle layers of V1 and V2 and contrast sensitivity is different between V1 and V2.	There is a difference in contrast sensitivity between V1 and V2 but there is no evidence for contrast variations as a function of cortical depth	Spatial deconvolution model that they adapted from Markuerkiaga et al. (2016) should be extended to other brain areas with different vascular structure, different experimental designs and imaging sequences
Muckli et al., 2015	GE-EPI	Magnetom 7T Siemens	2	0.8 mm^3^ iso	If we mask feedforward input to V1 area, any activity in that area will be result of feedback signals from other cortical areas	Non-stimulated V1 receives cortical feedback information to superficial layers	Non-BOLD sequences (e.g., VASO, ASL, etc.) can be used to purify data from venous artifacts in superficial layers
Raemaekers et al., 2014	EPI	Philips Achieva 7T	1.5	2 × 1.979 × 1.979 mm	Voxels that are in different fieldmaps but represent the same portion of the visual field would be expected to have highly correlated whereas voxels that are distant from each other will show less connectivity	Enhanced connectivity between same topographic locations in fieldmaps of V1, V2, and V3. Enhanced connectivity to the contralateral functional homolog	Connectivity among fieldmaps of V3a, V4, MT and parietal areas should be investigated.
Scheeringa et al., 2016	3D EPI	Magnetom Trio Tim 3T Siemens	2.3	0.75 mm^3^ iso	If different frequency bands show a distinctive relation with the laminar-resolved BOLD signal by combining data from simultaneously recorded EEG and fMRI from early visual cortex	Gamma band EEG power correlates positively with the superficial layers’ BOLD signal and beta-power is negatively correlated to deep layer BOLD and alpha power to both deep and superficial layer BOLD	Functional connectivity between FPN parts of the frontal cortex and visual areas can be assessed

## Author contributions

PD wrote the review article. GD and KV revised and provided feedback on the content of the manuscript. All authors contributed to the article and approved the submitted version.
